# The Relationship of Previous Training and Experience of Journal Peer Reviewers to Subsequent Review Quality

**DOI:** 10.1371/journal.pmed.0040040

**Published:** 2007-01-30

**Authors:** Michael L Callaham, John Tercier

**Affiliations:** 1 Division of Emergency Medicine, University of California, San Francisco, United States of America; 2 Department of Sociology, University of Lancaster, Lancaster, United Kingdom; Cincinnati Childrens Hospital, United States of America

## Abstract

**Background:**

Peer review is considered crucial to the selection and publication of quality science, but very little is known about the previous experiences and training that might identify high-quality peer reviewers. The reviewer selection processes of most journals, and thus the qualifications of their reviewers, are ill defined. More objective selection of peer reviewers might improve the journal peer review process and thus the quality of published science.

**Methods and Findings:**

306 experienced reviewers (71% of all those associated with a specialty journal) completed a survey of past training and experiences postulated to improve peer review skills. Reviewers performed 2,856 reviews of 1,484 separate manuscripts during a four-year study period, all prospectively rated on a standardized quality scale by editors. Multivariable analysis revealed that most variables, including academic rank, formal training in critical appraisal or statistics, or status as principal investigator of a grant, failed to predict performance of higher-quality reviews. The only significant predictors of quality were working in a university-operated hospital versus other teaching environment and relative youth (under ten years of experience after finishing training). Being on an editorial board and doing formal grant (study section) review were each predictors for only one of our two comparisons. However, the predictive power of all variables was weak.

**Conclusions:**

Our study confirms that there are no easily identifiable types of formal training or experience that predict reviewer performance. Skill in scientific peer review may be as ill defined and hard to impart as is “common sense.” Without a better understanding of those skills, it seems unlikely journals and editors will be successful in systematically improving their selection of reviewers. This inability to predict performance makes it imperative that all but the smallest journals implement routine review ratings systems to routinely monitor the quality of their reviews (and thus the quality of the science they publish).

## Abstract 2

A survey of experienced reviewers, asked about training they had received in peer review, found there are no easily identifiable types of formal training and experience that predict reviewer performance.

## Editors' Summary

**Background.:**

When medical researchers have concluded their research and written it up, the next step is to get it published as an article in a journal, so that the findings can be circulated widely. These published findings help determine subsequent research and clinical use. The editors of reputable journals, including *PLoS Medicine,* have to decide whether the articles sent to them are of good quality and accurate and whether they will be of interest to the readers of their journal. To do this they need to obtain specialist advice, so they contact experts in the topic of the research article and ask them to write reports. This is the process of scientific peer review, and the experts who write such reports are known as “peer reviewers.” Although the editors make the final decision, the advice and criticism of these peer reviewers to the editors is essential in making decisions on publication, and usually in requiring authors to make changes to their manuscript. The contribution that peer reviewers have made to the article by the time it is finally published may, therefore, be quite considerable.

Although peer review is accepted as a key part of the process for the publishing of medical research, many people have argued that there are flaws in the system. For example, there may be an element of luck involved; one author might find their paper being reviewed by a reviewer who is biased against the approach they have adopted or who is a very critical person by nature, and another author may have the good fortune to have their work considered by someone who is much more favorably disposed toward their work. Some reviewers are more knowledgeable and thorough in their work than others. The editors of medical journals try to take in account such biases and quality factors in their choice of peer reviewers or when assessing the reviews. Some journals have run training courses for experts who review for them regularly to try to make the standard of peer review as high as possible.

**Why Was This Study Done?:**

It is hard for journal editors to know who will make a good peer reviewer, and there is no proven system for choosing them. The authors of this study wanted to identify the previous experiences and training that make up the background of good peer reviewers and compare them with the quality of the reviews provided. This would help journal editors select good people for the task in future, and as a result will affect the quality of science they publish for readers, including other researchers.

**What Did the Researchers Do and Find?:**

The authors contacted all the regular reviewers from one specialist journal *(Annals of Emergency Medicine).* A total of 306 of these experienced reviewers (71% of all those associated with the journal) completed a survey of past training and experiences that might be expected to improve peer review skills. These reviewers had done 2,856 reviews of 1,484 separate manuscripts during a four-year study period, and during this time the quality of the reviews had been rated by the journal's editors. Surprisingly, most variables, including academic rank, formal training in critical appraisal or statistics, or status as principal investigator of a grant, failed to predict performance of higher-quality reviews. The only significant predictors of quality were working in a university-operated hospital versus other teaching environment and relative youth (under ten years of experience after finishing training), and even these were only weak predictors.

**What Do These Findings Mean?:**

This study suggest that there are no easily identifiable types of formal training or experience that predict peer reviewer performance, although it is clear that some reviewers (and reviews) are better than others. The authors suggest that it is essential therefore that journals routinely monitor the quality of reviews submitted to them to ensure they are getting good advice (a practice that is not universal).

**Additional Information.**

Please access these Web sites via the online version of this summary at http://dx.doi.org/doi:10.1371/journal.pmed.0040040

• WAME is an association of editors from many countries who seek to foster international cooperation among editors of peer-reviewed medical journals

• The Fifth International Congress on Peer Review and Biomedical Publication is one of a series of conferences on peer review

• The *PLoS Medicine* guidelines for reviewers outline what we look for in a review

• The Council of Science Editors promotes ethical scientific publishing practices

• An editorial also published in this issue of *PLoS Medicine* discusses the peer review process further

## Introduction

Most authors and editors would agree that the expertise of those who perform peer reviews for scientific journals has a lot to do with the quality of what is disseminated to the scientific community to become the foundation of future research [1]. Nonetheless, despite 20 years of research presented at five International Conferences on Peer Review, there has been surprisingly little study of what training and qualities are necessary to function as a proficient scientific reviewer [2]. Even less is known about how peer reviewers should be selected, and yet all journals routinely appoint new reviewers whose true quality is often revealed only after a number of reviews.

It would be useful to be able to predict the likelihood of success of a peer reviewer from information readily available from a curriculum vitae or a brief survey, before they were ever appointed to this post. This mechanism would allow editors and journals to most efficiently develop strategies to recruit the best reviewers. However, only four previous studies have attempted to determine whether some combination of peer reviewer experience could predict the quality of their subsequent reviews; these studies were relatively limited in size (most examining only a few hundred reviews or less), and were often a subanalysis of a study of some other intervention (such as blinding reviewers) [3–6]*.*

We therefore conducted a study of a larger group of peer reviewers, reviews, and possible contributors to performance, using as an outcome measure a standardized quality rating already in long use, with the hypothesis that some combination of reviewer training and experience could be identified that predicted subsequent production of high-quality reviews.

## Methods

*Annals of Emergency Medicine* is the leading journal in the specialty of emergency medicine and ranks in the top 11% among 5,876 science and medical journals listed by the ISI in frequency of citations [7]. All reviewers at this journal are blinded as to the authors and institution of papers they are reviewing.

For over fifteen years every review at this journal has been rated for quality by an editor, based on a predefined 5-point score that has been shown to primarily reflect review quality [8]. Six components of a quality review are formally defined, and editors are asked to combine assessments of all of them into their single global quality score. By definition, scores of 1 or 2 are unsatisfactory, and reviewers who regularly perform at this level are no longer utilized, thus producing a reviewer pool that produces consistently good reviews. Reviews rated 3 are defined as satisfactory, 4 as superior, and 5 outstanding (“hard to improve”). This score system is highly similar to the global rating component of the system reported and validated by van Rooyen [9]. Reviewers are selected by editors for each manuscript based on the reviewer's schedule and availability, how well their expertise matches the topic of the submitted paper, and the quality of their past reviews. All three of these factors affect the volume of reviews done by a particular reviewer.

All permanent reviewers who had completed reviews during January 2002 to December 2005 were eligible for entry into this study and were invited to participate. If they consented, they were asked to complete a survey of their background and training in skills relevant to peer review and critical appraisal (Table 1). Senior editorial board members and one-time guest reviewers were excluded. The survey items were derived from those previously reported in studies of this topic [3–6], plus those hypothesized by researchers and editors at the Peer Review Congress in Barcelona in 2001, as well as our own editorial board, as being likely to contribute to performance as a good reviewer. Periodic surveys of reviewer background are conducted every four or five years at this journal, and served as pilots to develop the current survey. All reviewing at this journal is done electronically via a Web-based system, so email provides the most reliable access, is constantly kept up to date, and was the contact method used. For those who did not respond to the first email request, two more follow-up requests occurred at one-month intervals in early 2005. All reviewer scores during the study period were used for analysis, after individual identifiers for reviewers had been removed. The study was approved by the Committee on Human Research of the University of California San Francisco.

**Table 1:**
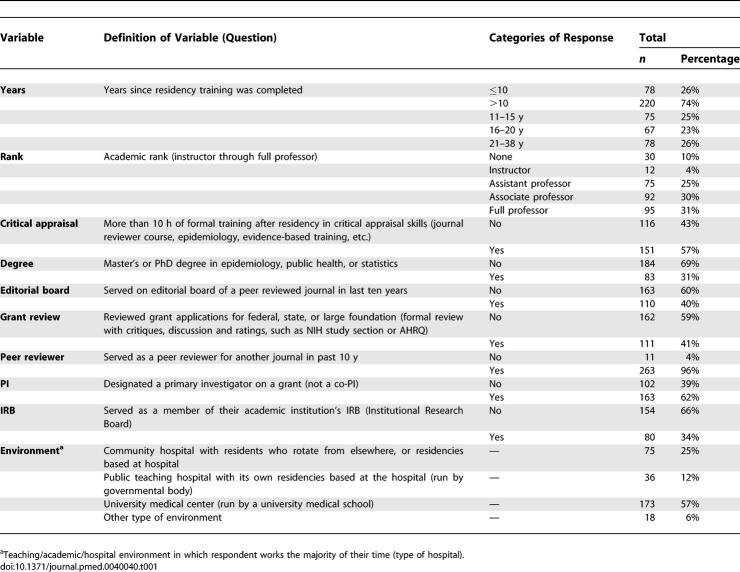
Questions on Survey and Response Rate

Two separate methods of identifying different levels of quality were analyzed because we felt that both could be useful metrics for editors. In the first (all reviews), review scores of 3 and above (satisfactory to outstanding) were compared to 1 or 2 (unsatisfactory), separating reviews into acceptable versus unacceptable groups. This distinction could be useful for a journal with difficulty in recruiting sufficient peer reviewers, a common dilemma in many small journals. In the second outcome (satisfactory reviews only), scores of 4 or 5 were compared to 3, thus separating the reviews into excellent versus satisfactory. This distinction could be useful for a journal with far more potential applicants than needed. Univariate GEE (generalized estimating equation) models were used to predict the quality of a review based on reviewer characteristics for either of these two outcomes. These were models for binomially distributed outcomes and used a logit link function. The models used an exchangeable working correlational structure for association of reviews within reviewers.

Both univariate and multivariable analyses were employed, but major conclusions in this paper are based on the multivariable analysis, which controlled for confounders. Since all the survey variables were chosen because of their logical connection to better scores, all were included in the multivariable GEE model. Analyses were carried out separately using both the aggregate review score for each reviewer, and the individual review score as the unit of analysis. The scores of individual reviews are predefined, validated, unambiguous, and easy to interpret. By comparison, any decision as to what ranges of scores constitute a particular level of performance for an aggregate score for a reviewer is arbitrary, and would be debated and disputed by readers, reviewers, and editors. Furthermore, even reviewers with excellent mean aggregate scores can and do produce individual reviews of poorer quality. We therefore chose to employ the individual review as the default unit of analysis, but also compared this method to that of using the aggregate review score. In the analysis by aggregate reviewer scores, a weight variable was used which is the reciprocal of the variance of the reviewer's mean score. For purposes of discussion (especially in the univariate analyses), a *p*-value of less than 0.10 was considered a trend (although nonsignificant), and less than 0.05 significant. All analyses were carried out in SAS, Version 9.1 (SAS, http://www.sas.com).

## Results

At the time of the survey there were 460 reviewers in the journal's pool of permanent reviewers. (Reviewers invited only once as a “guest” to review a particular manuscript were not included.) Of this number, 30 reviewers had performed no reviews during the study period and were excluded, leaving 430 who were sent the survey.

A final of 308 reviewers (72%) consented to participate and returned a completed survey instrument. Two reviewers submitted surveys with no identifier on them, which were excluded. The remaining 306 reviewers constitute the subjects in this study; they completed a total of 2,856 reviews of 1,484 separate manuscripts during the study period (71% of all journal reviews), with a mean score of 3.6 (median, 3.7; interquartile range [IQR], 0.7; standard deviation [SD], 0.7) by a total of 32 editors. The mean number of reviews per reviewer was 9.4 (median, 8; IQR, 8; SD, 7.7); the range was one review only (6% of all reviewers) to 46 reviews (0.3%). 25 reviews were rated 1, 164 rated 2, 717 rated 3, 1,296 rated 4, and 654 rated 5.

The 124 nonresponding reviewers had conducted 1,165 reviews for a mean 4.4 each (median, 3; IQR, 3; SD, 4.3) and a range of one review only (21% of all reviewers) to 27 (1% of reviewers). The mean quality score of nonresponders was 3.6 (median, 3.8; IQR, 1; SD, 0.8). There was no difference in the mean scores of reviews performed by responders versus nonresponders.

Table 1 summarizes the number and distribution of reviewer responses to the questions. The respondents were an experienced group with a broad range of training experiences; they averaged 15.7 years since residency training, 61% were associate or full professors, 57% had undergone formal critical appraisal training, 31% had a degree in epidemiology or statistics, 40% were on a journal editorial board, 41% had performed formal high-level grant review, 62% had been principal investigator on a grant, and 34% had served on an institutional review board (IRB). 57% practiced in a university-owned and -operated teaching hospital, as compared to other teaching environments.

Results of the univariate analysis are shown in Tables 2 and 3, and do not control for confounders. Most of the variables were not associated with a usefully large odds ratio at a statistically significant level. For the outcome of an acceptable versus unacceptable review, younger age (experience ≤10 y, odds ratio [OR], 1.66) predicted a better review, as did university versus all other types of environment (OR, 2.14, Table 2). Almost paradoxically, being a peer reviewer for another journal (OR, 0.24) predicted poorer reviews, and there was a similar nonsignificant trend for being on an IRB (OR, 0.67). For excellent versus satisfactory reviews, younger age (especially <10 y after training) (OR, 1.31), being on an editorial board (OR, 1.69), and university versus other environment (OR, 1.43) again predicted better-quality reviews (Table 3). However, being on an IRB again predicted poorer scores (OR, 0.69). In both tables, almost none of the ORs exceeded 2, so the effect was not large.

**Table 2:**
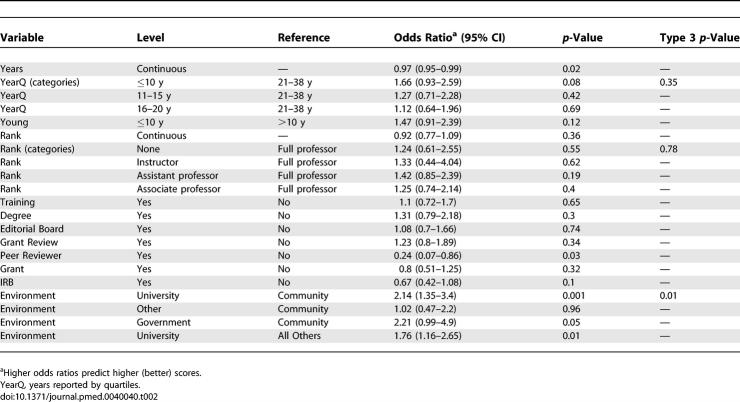
Relationship of Experience and Training to Quality Ratings, Univariate Analysis: Prediction of Acceptable Versus Unacceptable Review (Scores 3, 4, 5 Versus 1, 2)

**Table 3:**
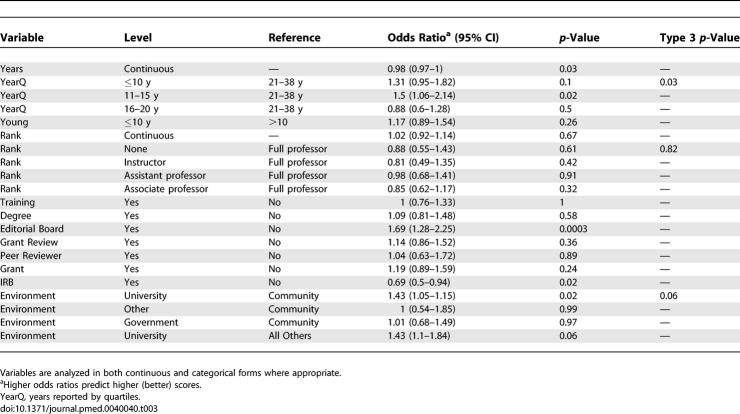
Relationship of Experience and Training to Quality Ratings, Univariate Analysis: Prediction of Excellent Versus Satisfactory Review (Scores 4, 5 Versus 3)

The multivariable logistic model (Tables 4 and 5) demonstrated that for predicting acceptable versus unacceptable reviews when controlled for the other variables, experience with grant review (OR, 1.89) and university environment (OR, 1.85) were associated with better reviews. There was also a similar nonsignificant trend (*p* = 0.09) for holding an advanced statistics degree. Being on an IRB was associated with worse reviews (OR, 0.60). For excellent versus satisfactory reviews, being on an editorial board (OR, 1.79) and university versus other teaching environment (OR, 1.42) were associated with better reviews; being on an IRB was associated with poorer reviews (OR, 0.72). None of the other experience or training variables predicted outcome. Even for variables with significant ORs, however, the predictive power of the model was poor, with an area under the curve of 0.52 for the model in Table 4 and 0.53 for the model in Table 5—not much better than chance.

**Table 4:**
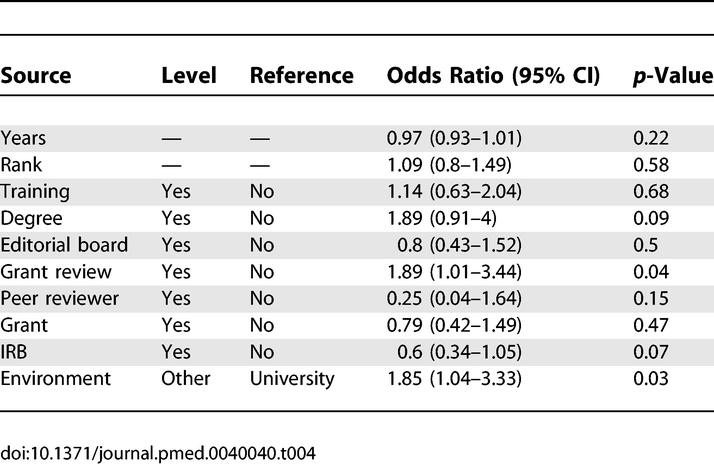
Relationship of Experience and Training to Quality Ratings, Multivariable Model: Acceptable Versus Unacceptable Review (Scores 3, 4, 5 Versus 1, 2)

**Table 5:**
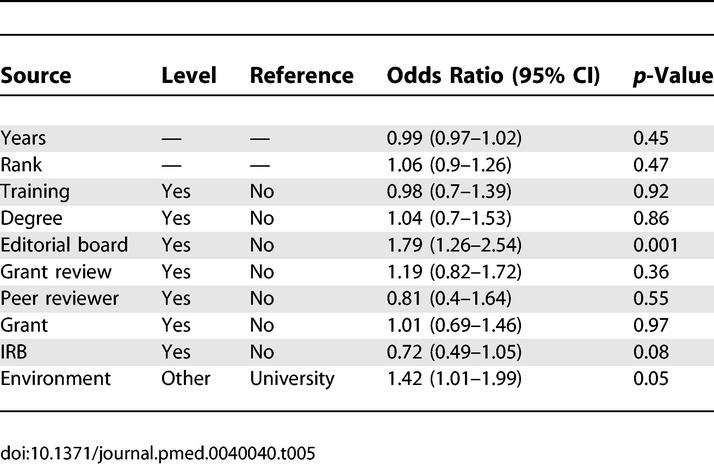
Relationship of Experience and Training to Quality Ratings, Multivariable Model: Excellent Versus Satisfactory Review (Scores 4, 5 Versus 3)

## Discussion

Our results show that, unfortunately, almost none of the experiences and training that might logically be thought to make for a high-quality reviewer (such as training in critical appraisal, academic rank, having been a funded primary investigator, serving on an IRB, etc.) actually predict subsequent performance of higher-quality reviews (Tables 2–5). The multivariable analysis (which controlled for confounders) showed that comparing acceptable versus unacceptable reviews, having participated in grant review, and university environment predicted a better review; there was a nonsignificant trend in favor of a degree in statistics. None of the other factors were predictive, except for serving on an IRB, which paradoxically was associated with lower-quality reviews. Using the outcome of excellent versus satisfactory reviews, only serving on an editorial board and university environment were associated with better-quality reviews. Again, IRB service was paradoxically associated with worse scores.

Most importantly, most of the ORs were less than 2, and even when our model produced ORs of 2 or more and significant *p*-values, the area under the curve was barely better than chance alone, demonstrating the lack of usefulness of these criteria in the real world.

Our study involved a large number of reviewers and reviews. It examined a larger number of types of training and experience that might be expected to improve reviewing skills, compared to prior studies. Reviews were rated by 32 editors, as compared to four or fewer previously [4–6]. It is the first study we are aware of that involved a standardized quality rating score long in use at the studied journal, rather than a newly developed score not previously employed. Additionally, we strove for practicality; all our data could be easily provided by a potential reviewer. We deliberately avoided extracting data from curricula vitae (such as authorship sequences on publications) because of the extra work involved for journal staff and the variability in interpretation of the data provided in that format. (Evans found a kappa of only 0.40 in trying to categorize publications listed in curricula vitae as original research or not [4].) We also deliberately avoided qualities so subjective as to be nonreproducible, such as judgment by single editors that reviewers were “well known,” “leaders in the field,” etc. We used a single global rating scale because its simplicity should enhance editor compliance, and because prior studies demonstrated no significant benefit to use of additional subscales [4,9]. We also avoided arbitrary definitions of reviewer quality based on ranges of mean scores, since many different definitions could be defended as logical and because even reviewers with excellent mean aggregate scores can produce individual reviews of poorer quality. Instead we reported the predictive power for quality by review (prediction of the quality of “the next review”), in categories useful to editors (namely, acceptable versus unacceptable reviews, and excellent versus satisfactory reviews). However, the results were not changed when we used the mean aggregate review scores of each reviewer as the unit of analysis.

Most authors and editors would probably agree that the quality of peer review is crucial to selecting and publishing the best science, but remarkably little study has been conducted to determine how to identify good reviewers. A search of PubMed since 1966 using the MeSH keywords “Peer Review/Research” and “Publication” or “Periodicals” identified only four prior studies on this topic, whose findings are summarized in Table 6. Stossel first reported on the quality of a year's worth of reviews performed at the *Journal of Clinical Investigation* in 1983 [3]. Reviews were judged by editors on a three-point scale not used in any other studies, and information was collected on reviewer's academic rank and “reputation as a leader in the field” (determined in some unspecified fashion by an unspecified subset of the editors). Review quality was proportionally lowest in the “high status” reviewer group (high rank and leadership reputation), who also had the highest proportion of refusals to review.

**Table 6:**
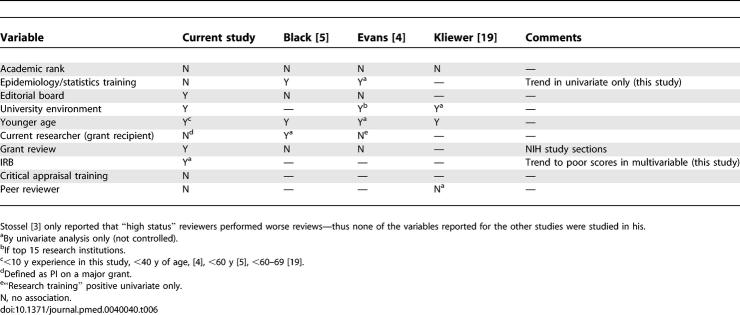
Summary of Variables Studied in Published Analyses

In a substudy of a randomized controlled trial of reviewer blinding, Evans reported on data from the curricula vitae of 201 internist reviewers of 131 manuscripts using a newly devised (and unvalidated) rating system with nine components as well as a global score [4]. More than half of reviews were of very poor or only medium quality; only three editors performed the ratings. When confounders were controlled for, only age below 40 years old and whether they were from one of the top 15 US academic instructions (rated by federal research dollars) predicted a good-quality review. Editorial board membership, gender, and National Institutes of Health study section membership did not, nor did being a prolific author or holding a senior leadership position. Training in research methods showed a nonsignificant trend for better reviews, whereas more senior academic rank showed a nonsignificant trend toward worse, but both effects disappeared in the multivariable model. A reviewer under 40 years of age, at a top academic institution, personally known to the editor choosing the review, and blinded to the authors' identity, had a 87% chance of producing a good review, whereas if none of these characteristics was present the chance was only 7%. Only 4% of reviewers met all the criteria in this model.

Black reported a subanalysis of a randomized controlled trial of blinding [5]. He evaluated the characteristics determining quality reviews of 420 manuscripts at the *BMJ,* rated by four editors using a newly developed but validated seven-component scale with a global summary rating very similar to the one we used [9]. A chief focus of the study was the association of quality with self-reported time spent on the review, but logistic regression showed that this had no predictive value, and only younger age (<40 y) and training in epidemiology or statistics were positively associated with review quality. However, these variables could explain only 2% of the variance in quality. Academic appointment, current research investigator status, publication experience, membership in a research funding body, and editorial board membership were not associated with quality.

Kliewer examined only limited demographic characteristics (no data on any training or analytic experience) versus review scores of radiology journal reviewers for one year, using a unique and unvalidated four-point quality score assigned by four or five editors, and reporting correlations only [6]. Review scores were highly (and negatively) correlated with age, with the largest dropoff at age 60 years. Scores were also lower for those practicing outside an academic environment; they found no significant association with gender, years of reviewing, or academic rank.

A comparison of our results with those of previous studies reveals a good deal of variation in the variables studied, and few common themes as to what is related to quality (based on multivariable analyses that controlled for confounding variables, Table 6). Many of the characteristics that seem logical have not in fact been shown to predict performance (e.g., academic rank, grant review). Two studies found (undefined) epidemiology “interest” or “expertise” to be predictive; we required a degree in statistics or epidemiology as our definition of this expertise and found a nonsignificant trend in one outcome (acceptable versus unacceptable review, Table 4) but not in the other (excellent versus satisfactory review, Table 5). Two prior studies did not find a benefit to editorial board membership, but we did in the outcome of excellent versus satisfactory. University environment, although defined in differing ways, was generally found by all to predict better quality (see Table 6). Another common theme was younger age, although our definition of ten years of experience and Evans' similar criterion of age less than 40 years, differ from the cutoff of less than 60 years of age the two other studies, and the rates of decline at various ages varied widely. Being the principal investigator on a grant was not beneficial, nor did most studies find that being on a study section for grant review was helpful. Ours was the only study to examine IRB membership, which demonstrated a nonsignificant trend toward lower quality in both of our outcomes. As for the reasons for these associations, we can only speculate. Editorial board membership and university teaching environment seem logically associated with better reviewing skills. It is less obvious why younger age should predict better reviews, although younger reviewers may be more motivated, spend more time, and have fewer competing obligations than those more advanced in their careers. We speculated that IRB membership may be a marker of more senior academic status, similar to increased age, and thus associated with lower scores, but this explanation was not supported by the multivariable analysis.

### Limitations

Our subjects were reviewers successful enough to be retained by the journal, and thus not a representative sample of all those who might apply to become reviewers. (Only 189 [7%] of all the reviews had an unsatisfactory score [1 or 2].) None of them were newly appointed to the journal. We would have liked to assess reviewers completely new to the journal, but the annual number of recruits is small and many of them do not perform substantial numbers of reviews, making such an analysis logistically impossible. Reviewers who returned the survey might not be similar in experience or performance to those who did not respond, although we did have a high rate of response (over 70%) and the quality score of respondents did not differ from that of those who responded to the survey. However, nonresponders did have a review volume less than half that of responders. It is possible that our study population may under-represent poor or less committed reviewers, but when we tested the performance of the multivariable model in predicting review outcome, it was the same for both groups.

All reviewers in this study came from a single-specialty peer review journal (as in previous studies), but our reviewers had appointments at virtually every US medical school and a broad variety of backgrounds and training. This particular reviewer population has been well studied in the past, and has performed similarly to journal reviewers from other specialties. For example, their ability to detect deliberately introduced flaws in a manuscript was very similar to that in a large general medicine journal and a small Scandinavian language journal [10–12]. Studies have also been done on this population to determine the impact (or lack of impact) of different forms of reviewer training [13–16], and the training results reported earlier in our journal's reviewer pool have recently been replicated by a large general medicine journal on their own reviewers [12]. Because this reviewer population has been more thoroughly compared to those of other journals than reviewers in previous reports, we believe that our results are likely to be generalizable to peer reviewers in other specialties and journals.

Our outcome measure was scores on a previously reported global rating scale of review quality [8]. There is only one validated review rating scale in existence [9], but we did not use that exact scale because it would have required changing a 15-year practice at our journal, and because it required the extra labor of seven additional subratings that we believed would reduce editorial compliance. Furthermore, the authors of that scale reported that the subscales did not vary in predictive ability from the overall global scale, and that their journal now uses only the latter. A similar lack of improvement with subscales was reported by Evans [4]. We therefore chose the simpler method, which is more practical and, although not identical to van Rooyen's, is probably highly similar.

### Conclusions

Our study confirms and expands the prior literature by examining experience and training that seem logically relevant to the development of good review skills. It confirmed prior findings that more experienced reviewers (>10 y after residency in our study) perform lower-quality reviews than do younger ones, and found that only editorial board experience, grant review, and working in a university hospital environment (versus other types of teaching environments) were associated with better-quality reviews in multivariable analysis. However, even these predictors were weak, with a small area under the curve in our study and poor predictive power in the other studies that reported on that measure [4,5]. None were powerful enough to be useful in selecting reviewers.

Building on the few previous reports, our findings suggest that it will not be easy to identify types of formal training and experience that predict reviewer performance, and indeed there may be none. It has not yet even been demonstrated that the qualities that make a good reviewer can be taught; the studies done so far show no effect of conventional reviewer training [12,14,16]. Instead, reviewer performance may be based on qualities for which we have not as yet determined good methods of identification and measurement, such as skepticism, thoroughness, motivation, inherent talent in detecting design weaknesses, etc. Skill in scientific peer review may be as ill defined and hard to impart as is “common sense,” particularly if reviewers' decision-making is based on intuitive recognition of complex patterns of “quality” in the manuscript and not on rational analysis of simple components [17]. Clearly we do not yet understand the crucial elements that shape a good reviewer. Without that information it seems unlikely that journals and editors will be successful in screening or designing training for those qualities—a crucial limitation in the peer review process.

The reviewer selection processes of most journals, and thus the qualifications of their reviewers, are ill defined [2,18]. More objective selection of peer reviewers might improve the journal peer review process and thus the quality of published science, but the data presented here confirm that commonly available information about reviewer training and experience does not predict subsequent performance. This being the case, it becomes all the more imperative that all journals with more than a very small number of editors and/or reviewers develop and implement rating systems for all reviews, and monitor the performance of reviewers on a regular basis. Without such a quality control measure, there is no way to know the quality of appointed reviewers and thus of the reviews that are used to assess the contents of the journal—yet many journals have no such quality control mechanism, nor can a reader readily determine if such a mechanism is in place. (In the only journal to report on this topic, a newly implemented rating system revealed that only 43% of their reviewers performed good or excellent reviews, compared to 33% fair and 24% poor or extremely poor [4].)

There are two possible approaches for future research. One could involve performing more studies similar to ours with more complex analyses, covering multiple journals and many thousands of reviewers, and collecting data on many more variables that might predict quality performance. The disadvantage of this approach is that we do not know enough to do more than guess at relevant variables to assess, and even if a predictive model were found, the resulting tool might not be practical for use in the everyday life of journals. We predict that this approach would not be very productive.

A second approach would be to go back to the beginning, collaborating with experts in cognition and learning to identify and understand the specific analytic strategies and thought processes used in manuscript reviews conducted by high-quality reviewers. This would probably require initial qualitative research, which could generate new hypotheses about which easily identifiable characteristics of reviewers might be associated with quality review. (We can only hope there are such characteristics, and that detecting reviewer talent does not require the development of extensive special testing processes.) If we knew what cognitive approach(es) produced a good review, we might not only be able to identify in advance those who use that approach, but we might also be able to design educational interventions to strengthen those skills in all reviewers—something that has eluded us so far. The chief obstacle to this approach, as mentioned above, is that reviewers' decisions may be based on complex pattern recognition far more difficult to understand than simple, mechanistic problem solving.

## Abbreviations

GEE: generalized estimating equation
IQR: interquartile range
IRB: institutional review board
OR: odds ratio
SD: standard deviation
